# Maturity related metabolomic analysis of *Balanites aegyptiaca* fruits with in vitro and in silico cytotoxicity evaluation

**DOI:** 10.1038/s41598-025-15234-y

**Published:** 2025-08-23

**Authors:** Asmaa Abdelsalam, Ehab Mahran, Eslam T. Mohamed, Arezue Boroujerdi, Hebatallah Aly

**Affiliations:** 1 Botany and Microbiology Department, Faculty of Science, Helwan University, Cairo, 11795 Egypt; 2 CAMAG Chemical Products and Adsorption Technology AG, 4132 Muttenz, Switzerland; 3 Chemistry Department, Claflin University, Orangeburg, SC 29115 USA

**Keywords:** *Balanites aegyptiaca* (L.), Fruit maturation, NMR spectroscopy, Metabolomics, Anticancer, *J*-resolved NMR, Biochemistry, Plant sciences

## Abstract

*Balanites aegyptiaca* (L.) Delile, a medicinal tree, produces an edible fruit widely recognized in traditional medicine for its antidiabetic and liver-enhancing properties. This study investigated the metabolic changes occurring during fruit maturation using integrated nuclear magnetic resonance (NMR) metabolomics approaches, including 1D ^1^H (proton), 2D heteronuclear single quantum coherence (HSQC), and 2D *J*-resolved NMR spectroscopy. A total of forty-five metabolites were identified and quantified, with key metabolites characterizing each maturation stage. Metabolic profiling indicated that immature fruits were characterized by elevated concentrations of amino acids, alkaloids, and organic acids, while mature fruits predominantly accumulated monosaccharides. Chemometric analyses and hierarchical clustering confirmed a significant metabolic differentiation between immature and mature fruit stages. Pathway analysis identified significant alterations predominantly in starch–sucrose metabolism, pyruvate metabolism, and the citrate cycle during maturation. Cytotoxic evaluation revealed that polar extracts from immature fruits exhibited superior cytotoxic activity against hepatocellular carcinoma cells (IC_50_ = 117.7 µg/mL) compared to mature fruit extracts (IC_50_ = 270.4 µg/mL). Molecular docking analysis further demonstrated that metabolites upregulated in immature fruits, like theophylline, showed a strong binding affinity (− 5.317 kcal/mol) to the anti-apoptotic protein BCL-2, suggesting their potential role in apoptosis regulation. This study provides insights into the metabolic dynamics during *Balanites aegyptiaca* fruit maturation, highlighting the superior therapeutic potential and significant cytotoxic activity of immature fruits compared to traditionally utilized mature fruits.

**Supplementary Information:**

The online version contains supplementary material available at 10.1038/s41598-025-15234-y.

## Introduction

*Balanites aegyptiaca* (L.) Delile (*B. aegyptiaca*), commonly known as the desert date, is a drought-resistant tree species belonging to the family Zygophyllaceae. It is widely distributed across arid and semi-arid regions of Africa, the Middle East, and South Asia^1^. The plant, especially its fruit, holds a significant traditional value in folk medicine, where it has been used to treat a variety of ailments including diabetes, liver dysfunction, gastrointestinal disorders, and respiratory illnesses^2^. These therapeutic properties are largely attributed to its diverse array of bioactive compounds such as phenolic acids, flavonoids, alkaloids, and saponins which have demonstrated antioxidant, anti-inflammatory, antimicrobial, and anticancer activities^3^. Beyond its medicinal importance, the fruit is rich in essential nutrients, including vitamins, minerals, and carbohydrates^4^.

Fruit maturation is a highly dynamic process that encompasses significant genetic, physiological, and biochemical changes, shaping the fruit’s composition, quality, and functionality^5,6^. This maturation process is regulated by hormonal signaling molecules such as ethylene, which play a crucial role in coordinating the metabolic transitions associated with ripening. The metabolic transformations occurring during fruit maturation offer crucial insights into the biochemical pathways responsible for the development of flavor, texture, nutrients, and overall fruit quality^7^. The transition from the immature to the mature stage in fruits is marked by profound metabolic shifts, including changes in primary metabolites essential for growth and energy, as well as secondary metabolites that influence the fruit’s potential bioactivities^8,9^.

In arid zones such as south of Egypt, where *B. aegyptiaca* naturally grows, the growth environment is characterized by extreme conditions: prolonged drought, high solar radiation, and summer temperatures frequently exceeding 42 °C^10,11^. These conditions can lead to altered ripening patterns and stress-driven metabolic reprogramming. In this harsh habitat, accelerated fruit development and enhanced antioxidant responses are often observed as adaptive strategies to complete the reproductive cycle before critical stress thresholds are surpassed^12,13^. The distinct stress-induced metabolic pathways in medicinal desert plants remain largely unexplored, yet their investigation could provide critical insights into elucidating both the physiological and metabolomic adaptations to such extreme conditions and the pharmacological efficacy of the plant’s fruit.

Metabolic profiling offers a robust approach to unravel the complex biochemical networks of medicinal plants, enabling the identification and quantification of metabolites involved in the fruit maturation process. By employing different omics approaches, it is possible to understand the molecular physiology during fruit ripening^14^. 1D NMR experiments, such as ^1^H NMR, are predominantly utilized in metabolomic studies due to their capacity to deliver rapid and quantitative analyses of metabolite mixtures. However, the inherent complexity and peak overlap in 1D NMR spectra can obscure essential data, particularly in samples characterized by a high diversity of metabolites. 2D NMR spectra are used to enhance spectral resolution by distributing NMR signals across a secondary frequency dimension. This distribution not only clarifies overlapping peaks but can also facilitate the interpretation of coupling patterns and chemical shifts (for example, 2D *J*-resolved NMR)^15,16^. Although 1D and 2D NMR techniques provide excellent structural information and reproducibility, 2D NMR workflows typically require longer acquisition time, compared to other techniques^17^.

To gain a comprehensive understanding of the metabolomic mechanisms underlying fruit ripening in extremophile plants such as *B. aegyptiaca*, it is essential to integrate NMR-based metabolomics with advanced statistical and pathway analyses. In this study, the metabolic profiling of *B. aegyptiaca* fruit ripening under the extreme environmental conditions of Aswan in Egypt, an arid region characterized by intense and prolonged drought stress, was investigated. Using high-resolution NMR-based metabolomics, stage-specific metabolites were identified to delineate the associated biochemical pathways involved in the ripening process. Furthermore, cytotoxic activity of immature and mature fruit extracts was investigated through in vitro assays and in silico docking study.

## Materials and methods

### Plant material

The fruits of *B. aegyptiaca* were collected in June and August 2022 from the botanical garden of Aswan University in Egypt with geographical coordinates of 24.00° N and 32.86° E. Five trees, each representing one replicate, were randomly selected. Immature fruit was collected in June 2022, whereas the mature fruit was collected in August 2022. The identification of the plant was carried out through a detailed examination of the plant’s morphological characteristics, which were then compared to the literature^18^. The plant was further authenticated by Prof Hasnaa Hosni, professor of Plant Taxonomy at the Botany Department of Cairo University in Egypt and (Herbarium specimen No. HEU 011107) was kept at the herbarium of Faculty of Science, Helwan University in Egypt. The fruits collected for running the experiments were immediately immersed in liquid nitrogen and subsequently kept at − 80 °C.

### Metabolite extraction

The frozen fruits were lyophilized for 24 h, and then completely homogenized. Twenty mg of each replicate was extracted using methanol, chloroform, and water in the proportions of 2:2:1.8 (*V/V/V*) based on the dry mass and water loss ratio^19,20^. Each extract was centrifuged at 5000 rpm for 5 min and the upper aqueous layer (polar extract) was separated and dried under vacuum.

### NMR data collection, metabolite identification, and quantification

The dry extract was redissolved in 620 µL NMR buffer, comprising of 1 mM TMSP-d4 (deuterated trimethylsilylpropanoic acid), 100 mM sodium phosphate buffer, and 0.1% sodium azide in 99.9% D_2_O. NMR data was collected using 700 MHz Bruker Avance™ III spectrometer controlled by TopSpin 3.5 software. The ^1^H NMR spectra had a width of 16.0 ppm and 64K data points. During a 3-s recycling delay, on-resonance pre-saturation was employed to suppress the solvent. The initial increment of the pre-saturation nuclear Overhauser effect spectroscopy (pre-sat NOESY) spectra was obtained using a total of 120 scans, including 4 dummy scans, with a relaxation delay of 3 s and pre-saturation at the residual water frequency. The 90° pulse width for each sample was determined using the automatic pulse calculation experiment (pulsecal) feature in TopSpin 3.5 software (Bruker BioSpin, Billerica, MA). The data collection involved the use of a Bruker hsqcedetgpsisp 2.2 pulse sequence to acquire the ^1^H-^13^C HSQC data. The ^1^H resonance was detected in the F2 dimension, exhibiting a spectral width of 16 ppm, whereas the ^13^C resonance in the F1 dimension displayed a spectral width of 252 ppm. The acquisition of the 2D *J*-resolved NMR spectra involved 12 scans and 16 dummy scans with TD of 8192 for F2 and 40 for F1 dimension. The spectral widths used were 16 ppm in F2 dimension and 78 Hz in F1 dimension. A relaxation time of 1.0 s was utilized. The windows function was assigned to SINE for both dimensions. The sine-bell shift (SSB) was set to 0. The raw 2D *J*-resolved spectra was processed (tilted and symmetrized with respect to F1 = 0) and projected using Mnova 9.1.0 software (Mestrelab Research, S.L., USA, https://mestrelab.com/). The processed spectra were extracted using Mnova Software Suite.

Identification of metabolites was initially conducted through a comparison of the 700 MHz ^1^H data obtained from polar extracts with the 700 MHz database available in the Chenomx NMR Suite (Edmonton, Alberta, Canada). Spectral peaks were fitted with the standards in the Chenomx database for identification. For peaks that exhibited overlap in the proton spectra, *J* coupling constants and ^1^H-^13^C HSQC cross peaks were cross-referenced with previously published ^1^H-^13^C HSQC data and data obtained from the online sources: Human Metabolome Database (HMDB) and Biological Magnetic Resonance Bank (BMRB).

Chenomx provides the concentration of each fitted metabolite in mM based on the proportionality of the metabolite’s peak area and proton equivalence to those of the internal standard, TMSP (1.0 mM). Use of a standard 1D pre-sat NOESY, which is not sensitive to small T1/T2 variation, along with controlled sample conditions (temperature and pH/buffer) ensures consistent relaxation behavior in most metabolites; thus, fitting experimental spectra to Chenomx’s library of standards yields valid metabolite concentrations.

### NMR data treatment and statistical and pathway analysis

Raw NMR spectra were processed using TopSpin 3.5 for alignment, baseline correction, phase correction, and chemical shift referencing to TMSP at 0.0 ppm. Spectral data were extracted from the 0.5–10.0 ppm region (excluding the water region at 4.733–4.833 ppm) with 0.01 ppm bucket widths using AMIX software 4.0 (Bruker BioSpin, Billerica, MA: https://www.bruker.com/en/products-and-solutions/mr/nmr-software/amix.html). The resulting bucket tables were exported to MetaboAnalyst 6.0^21^. Missing values were imputed as 1/5 of the minimum positive value for each metabolite to mitigate bias, followed by log transformation and autoscaling (mean-centered, unit variance) to normalize variance across features. Data preprocessing with univariate and multivariate analyses were then conducted.

The statistical analysis was based on the difference in the relative concentrations of the variables across the two fruit types. Principal component analysis (PCA) was applied to reduce dimensionality and visualize global metabolic variation. Sample clustering was assessed using Hotelling’s T^2^ ellipses (95% confidence intervals), and group separation significance was evaluated via permutational multivariate analysis of variance (PERMANOVA; 999 permutations). Partial least squares discriminant analysis (PLS-DA) was applied, with 95% confidence regions displayed to validate clustering robustness. Hierarchical clustering analysis (HCA) employed Euclidean distance as the similarity metric and Ward’s minimum variance algorithm for dendrogram construction.

Significant metabolites (based on concentration) between the two developmental stages were identified using volcano plot analysis, combining a fold change threshold of > 2 (log2|FC|> 1) and T-tests (*p* < 0.05). To preserve the biological relevance, FC ratios were calculated using raw, non-normalized data. Features meeting both thresholds were classified as significant. The top 24 significant metabolites were visualized as boxplots, depicting relative concentrations across developmental stages.

Pathway analysis was conducted using metabolite sets from the Kyoto Encyclopedia of Genes and Genomes (KEGG) human metabolic pathways. Significantly altered metabolites were mapped to pathways via MetaboAnalyst’s built-in library, and enrichment significance was assessed using a hypergeometric test. Pathway analysis was performed using *Arabidopsis thaliana* metabolic pathways as a reference. Pathway topology was weighted based on node centrality.

### Cytotoxic activity against hepatocellular carcinoma

The polar extracts of both fruit types were tested for their cytotoxic activity against hepatocellular carcinoma employing the colorimetric assay Sulforhodamine B (SRB). The hepatocellular carcinoma cell line was obtained from the Nawah Scientific Laboratory, Cairo, Egypt. Cells were maintained in DMEM medium supplemented with 100 mg/mL streptomycin, 100 units/mL penicillin, and 10% heat-inactivated fetal bovine serum in a humidified, 5% (*V/V*) CO_2_ atmosphere at 37 °C. The dried fruit extracts (*n* = 3) were solubilized in dimethyl sulfoxide (DMSO). Cell suspensions (100 µL, 5 × 10^3^ cells) were seeded in 96-well plates and incubated for 24 h. Cells were treated with 100 µL of medium containing *B. aegyptiaca* fruit extracts at various concentrations (0.0–400 μg/mL) for 72 h. Stock solutions were prepared in DMSO and serially diluted in the culture medium to ensure the final DMSO concentration in each well was maintained at 0.1% (v/v). The medium was replaced with 150 µL of 10% trichloroacetic acid (TCA) and incubated at 4 °C for 1 h. After TCA removal and rinsing with distilled water, 70 µL of 0.4% SRB solution was added, incubated for 10 min at room temperature, and protected from light. Paclitaxel was used as a positive control. The negative control consisted of untreated cells with culture medium containing DMSO. The blank contained medium and SRB reagent without cells. Post-rinsing with 1% acetic acid and air-drying preceded the absorbance measurement at 540 nm using a FLUOstar® Omega reader after SRB dissolution in 10 mM TRIS.

### Molecular docking study

Molecular docking was conducted to assess the binding interactions of metabolites from polar extracts of immature and mature *B. aegyptiaca* fruits with the anti-apoptotic protein BCL-2, using Schrödinger Maestro (version 2023-2, Schrödinger, LLC, New York, NY, USA) with the Glide module. The BCL-2 structure (PDB ID: 4LVT) was obtained from the Protein Data Bank (www.rcsb.org) and prepared using the Protein Preparation Wizard, which involved adding hydrogens, assigning bond orders, optimizing hydrogen-bond networks, removing non-essential water molecules beyond 5 Å from the active site, and minimizing with the OPLS4 force field to a root-mean-square deviation (RMSD) of 0.3 Å. From 45 statistically significant metabolites (fold change > 2, *p* < 0.05), 29 were selected for docking based on high/moderate abundance in immature fruits, reported anticancer activity or metabolic relevance to cancer, and structural feasibility (molecular weight ≤ 500 Da, suitable hydrogen bond donors/acceptors), as verified via PubChem (https://pubchem.ncbi.nlm.nih.gov). Metabolite structures were prepared using LigPrep, generating ionization states at pH 7.0 ± 2.0 with Epik, enumerating tautomers, and minimizing with OPLS4. A receptor grid (20 Å × 20 Å × 20 Å) was centered on the BCL-2 active site using Glide’s Receptor Grid Generation tool. Docking was performed in Glide Extra Precision (XP) mode, retaining up to 10 poses per ligand. The protocol was validated by redocking the co-crystallized ligand (RMSD < 2 Å). Docking scores (kcal/mol) and interactions (hydrogen bonds, salt bridges, π–cation, π–π stacking, hydrophobic contacts) were analyzed using Maestro’s Pose Viewer and visualized in PyMOL (version 2.5).

## Results and discussion

### Metabolomic analysis of the polar extracts

**Fig. 1:**
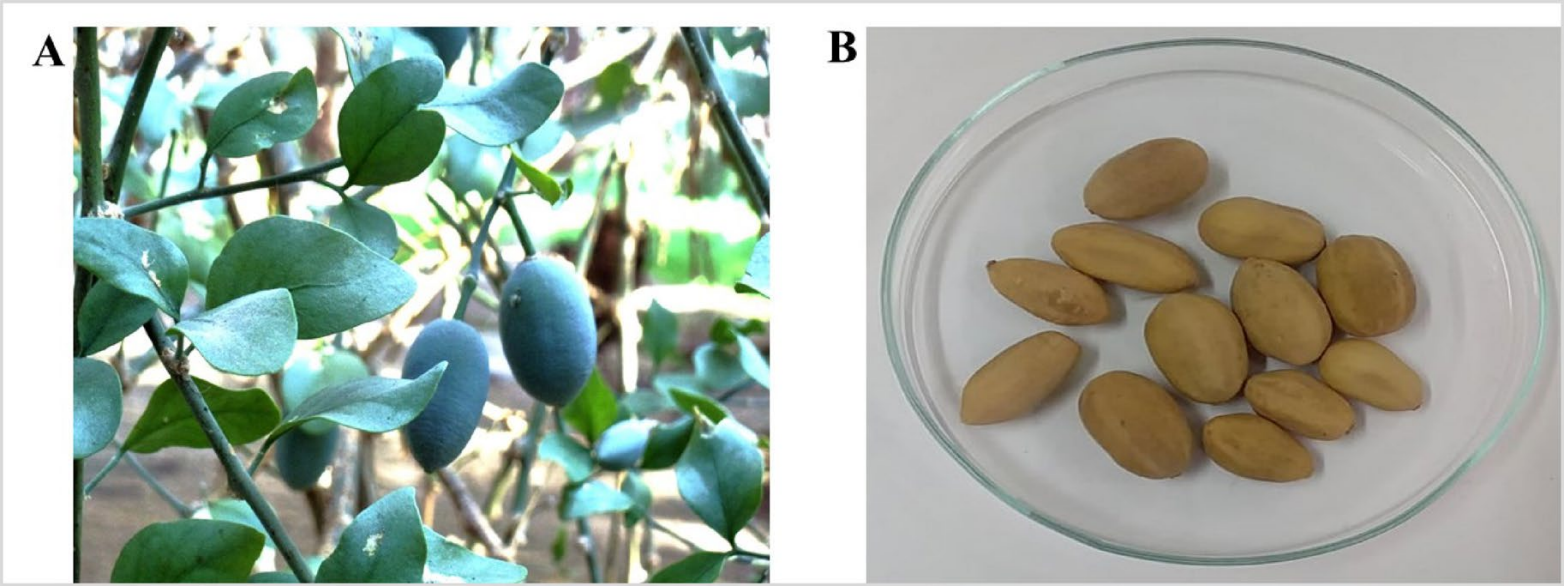
Fruit of the desert date, *B. aegyptiaca* collected from Aswan, Egypt. (**A**) Immature fruit and (**B**) Mature fruit.

The collected fruit was ellipsoid-shaped, measuring approximately 5.5 cm in length and 3.0 cm in diameter. The immature fruit exhibited a green color with a rough texture, while the mature fruit transitioned to a smooth, golden appearance, encased in a crispy exterior containing a sticky brown pulp (Fig. 1).

**Fig. 2:**
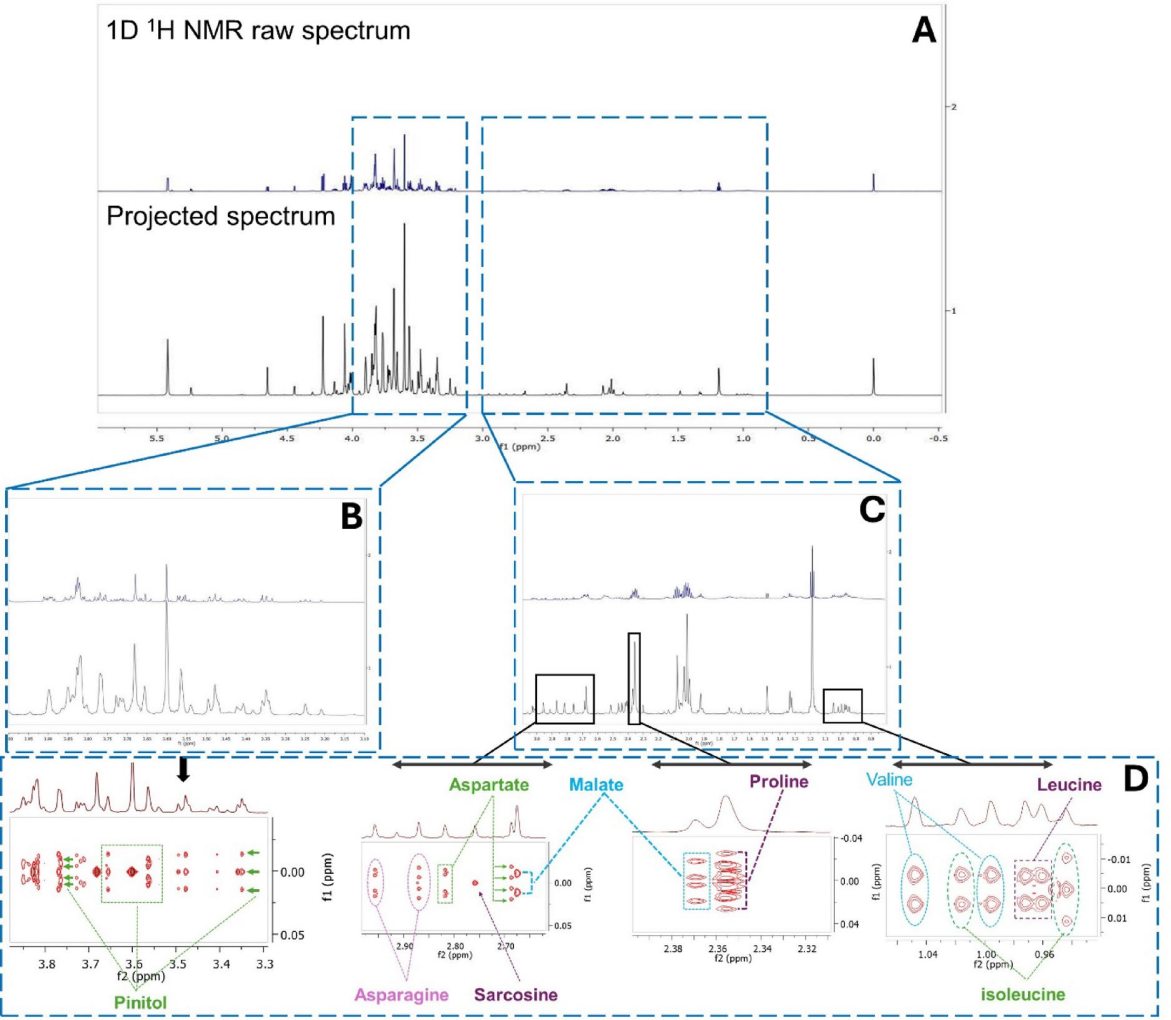
*J*-resolved NMR of *Balanites aegyptiaca* polar extract. Original and projected spectra in the region *δ*_H_ 0.0–6.0 ppm (**A**); expanded regions between *δ*_H_ 0.8–3.0 ppm (**B**); and *δ*_H_ 3.1–4.0 ppm (**C**) with their respective 2D *J*-resolved NMR spectra (**D**) of identified metabolites in each region.

The average extraction yields from the immature and mature fruit were 29.0% (5.8 mg) and 30.7% (6.1 mg), respectively. A total of forty-five metabolites were identified in the polar extracts of both immature and mature fruits (Table 1). While the 1D NMR data were helpful for annotating several metabolites (Figs. S1 and S2), the verification of certain metabolites was challenging due to signal congestion and overlapping peaks, particularly in narrow spectral regions. To address these challenges, HSQC (Fig. S3) and 2D *J*-resolved (*J*-res) NMR spectroscopy were employed. The projected spectra from the *J*-res experiments enabled the precise assignment of individual compounds, which were further confirmed by the 2D *J*-res spectra (Fig. 2). The identified metabolites span various chemical classes, with sugars being the dominant group in both mature and immature fruits (Fig. S4). In the densely populated sugar region (*δ*_H_ 3.0–5.5 ppm), six sugars and sugar alcohols were assigned based on their distinct signals, including the anomeric protons.

**Table 1:** Metabolites identified in the polar extract of *Balanites aegyptiaca* fruit.

	Compound	Weight (g/mol)	Formula	^13^C chemical shift (ppm) (functional group or specific C)	^1^H chemical shift (ppm) (functional group or specific H, multiplicity of peak)	Coupling constant *J* (Hz)	Immature fruit	Mature fruit
1	1,3-Dimethylurate	196.1634	C_7_H_8_N_4_O_3_	30.5 (CH_3_)	3.31 (CH_3_, s)	–	√	√
				32.8 (CH_3_)	3.44 (CH_3_, s)	–		
2	5-Hydroxytryptophan	220.2246	C_11_H_12_N_2_O_3_	105.0 (CH)	7.11 (CH, d)	0.5	√	√
				128.2 (CH)	7.26 (CH, s)	–		
3	γ-Aminobutyrate	103.1198	C_4_H_9_NO_2_	26.4 (CH_2_)	1.90 (CH_2_, m)	–	**√**	–
				37.0 (CH_2_)	2.28 (CH_2_, t)	7.3		
				42.0 (CH_2_)	2.99 (CH_2_, t)	7.4		
4	Alanine	89.0932	C_3_H_7_NO_2_	53.5 (C^α^)	3.78 (H^α^, q)	7.2, 7.2	√	√
				18.9 (C^β^)	1.46 (H^β^, d)			
5	Acetate	60.052	C_2_H_4_O_2_	26.1 (CH_3_)	1.92 (CH_3_, s)	–	√	–
6	Arginine	174.201	C_6_H_14_N_4_O_2_	26.6 (C^γ^)	1.64 (H^γ^, m)	–	√	√
				30.3 (C^β^)	1.92 (H^β^, m)	–		
				57.1 (C^α^)	3.75 (H^α^, t)	6.2		
7	Asparagine	132.1179	C_4_H_8_N_2_O_3_	54.2 (C^α^)	4.01 (H^α^, q)	4.2	√	–
				37.4 (C^β^)	2.95 (H^β^, dd)	4.1, 17.0		
					2.88 (H^β^, dd)	7.0, 17.0		
8	Aspartate	133.1027	C_4_H_7_NO_4_	54.9 (C^α^)	3.88 (H^α^, dd)	8.80, 2.7	√	√
				39.2 (C^β^)	2.66 (H^β^, dd)	17.7, 8.80		
9	Betaine	117.1463	C_5_H_11_NO_2_	69.2 (CH_2_)	3.90 (H^α^, s)	–	–	√
				56.2 (CH_3_)	3.26 (H^β^, s)	–		
10	Choline	104.1708	C_5_H_14_NO	58.6 (C^α^)	4.06 (H^α^_,_ m)	–	√	√
				56.8 (C^γ^)	3.21 (H^γ^, s)	–		
				70.4 (C^β^)	3.52 (H^β^, m)	–		
11	Citrate	192.1235	C_6_H_8_O_7_	48.4 (CH_2_)	2.52 (CH_2,_ d)	2.5	√	√
				48.4 (CH_2_)	2.65 (CH_2,_ d)	2.6		
12	Dimethylamine	45.0837	C_2_H_7_N	37.0 (CH_3_)	2.73 (CH_3_, s)	–	√	√
13	Erythritol	122.1198	C_4_H_10_O_4_	74.7 (CH)	3.66 (CH, m)	–	√	√
				65.3 (CH)	3.78 (CH, dd)	–		
14	Formate	46.0254	CH_2_O_2_	–	8.44 (CH, s)	–	√	√
15	Fructose	180.1559	C_6_H_12_O_6_	78.2 (^3^CH)	4.12 (^3^CH, dd)	–	√	√
				66.1 (^6^CH_2_)	4.02 (^6^CH_2_, dd)	12.7, 1.0		
				72.0 (^5^CH)	4.00 (^5^CH, m)	7.7, 5.4		
				66.6 (^1^CH)	3.56 (^1^CH, m)	–		
16	Glucose	180.1559	C_6_H_12_O_6_	98.9 (^1α^CH)	4.66 (^1α^CH, d)	7.8	√	√
				94.8 (^1β^CH)	5.24 (^1β^CH, d)	3.9		
				74.4 (^2α^CH)	3.53 (^2α^CH, m)	–		
				72.6 (^4^CH)	3.41 (^4^CH, m)	–		
				63.7 (^6^CH)	3.74 (^5β^CH, m)	–		
17	Glucose-6-P	260.1358	C_6_H_13_O_9_P	94.9 (CH)	5.20 (CH, d)	3.75	√	√
				98.9 (CH)	4.60 (CH, d)	8.0		
				65.4 (CH)	4.00 (CH, m)	–		
18	Glutamate	147.1293	C_5_H_9_NO_4_	29.7 (C^β^)	2.04 (H^β^, m)	–	–	√
				36.2 (C^γ^)	2.35 (H^γ^, m)	–		
				57.3 (C^α^)	3.74 (H^α^, dd)	7.1, 4.7		
19	Glutamine	146.1445	C_5_H_10_N_2_O_3_	33.7 (C^γ^)	2.46 (H^γ^, m)	–	√	√
				29.1 (C^β^)	2.14 (H^β^, m)	–		
				57.0 (C^α^)	3.78 (H^α^, t)	6.3		
20	Glycine	75.0666	C_2_H_5_NO_2_	44.3 (C^α^)	3.56 (H^α^, s)	–	√	–
21	Histamine	111.1451	C_5_H_9_N_3_	26.7 (CH_2_)	3.00 (CH_2_, t)	7.12	√	–
				41.8 (CH_2_)	3.29 (CH_2_, t)	7.12		
				119.0 (CH)	7.09 (CH, s)	–		
				138.7 (CH)	7. 91 (CH, s)	-		
22	Homoserine	119.1192	C_4_H_9_NO_3_	35.1 (C ^β^)	2.10 (H^β^, m)	–	√	√
23	Isoleucine	131.1729	C_6_H_13_NO_2_	26.8 (C^γ^)	1.29 (H^γ^, m)	–	√	–
				17.5 (C^γ^)	1.00 (H^γ^, d)	7.1		
				13.9 (C^δ^)	0.91 (H^δ^, t)	7.5		
24	Lactate	90.0779	C_3_H_6_O_3_	22.2 (CH_3_)	1.33 (CH_3_, d)	6.8	√	–
25	Leucine	131.1729	C_6_H_13_NO_2_	24.7 (C^δ^)	0.97 (H^δ^, d)	6.44	√	–
26	Lysine	146.1876	C_6_H_14_N_2_O_2_	32.5 (C^β^)	1.80 (H^β^, m)	–	√	–
				41.6 (C^ε^)	3.00 (H^ε^, t)	–		
				57.1 (C^α^)	3.70 (H^α^, t)	6.9		
27	Malate	134.0874	C_4_H_6_O_5_	45.2 (CH_2_)	2.36 (CH_2_, dd)	15.5, 10.3	√	√
				45.4 (CH_2_)	2.65 (CH_2_, dd)	15.5, 2.9		
28	Malonate	104.0615	C_3_H_4_O_4_	50.2 (C^β^)	3.12 (H^β^, s)	–	√	√
29	Methylamine	31.0571	CH_5_N	27.6 (CH_3_)	2.58 (CH_3_, s)	–	√	–
30	Oxypurinol	152.1109	C_5_H_4_N_4_O_2_	128.8 (CH)	8.2 (CH, s)	–	–	√
31	Pinitol	194.183	C_7_H_14_O_6_	62.3 (CH_3_)	3.60 (CH_3_, s)	9.8	√	√
				72.5 (^1^CH)	3.82 (^1^CH, m)	–		
				74.0 (^2^CH)	3.56 (^2^CH, dd)	–		
				85.8 (^3^CH)	3.35 (^3^CH, t)	9.8		
				74.8 (^4^CH)	3.35 (^4^CH, t)	4.0, 10.3		
				73.2 (^5^CH)	3.76 (^5^CH, dd)	4.0, 10.3		
				72.5 (^6^CH)	3.82 (^6^CH, m)	–		
32	Phenylalanine	165.1891	C_9_H_11_NO_2_	39.2 (C^β^)	3.09 (H^β^, dd)	7.0	√	–
				58.9 (C^α^)	3.99 (H^α^, dd)	6.3		
				132.1 (C^δ^)	7.31 (H^δ^, m)	–		
				130.4 (C^ζ^)	7.36 (H^ζ^, m)	–		
				31.8 (C^ε^)	7.41 (H^ε^, m)	–		
33	Proline	115.1305	C_5_H_9_NO_2_	64.2 (C^α^)	4.13 (H^α^, m)	–	√	√
				49.0 (C^δ^)	3.41 (H^δ^, m)	–		
				31.9 (C^β^)	2.36 (H^β^, m)	–		
34	Pyroglutamate	129.114	C_5_H_7_NO_3_	27.9 (C^γ^)	2.40 (H^γ^, m)	–	√	√
				60.9 (C^α^)	4.16 (H^α^, dd)	–		
35	Pyruvate	88.0621	C_3_H_4_O_3_	29.3 (CH_3_)	2.35 (CH_3_, s)	–	√	√
36	Sarcosine	89.0932	C_3_H_7_NO_2_	35.5 (CH_3_)	2.73 (CH_3_, s)	–	√	–
				53.4 (CH_2_)	3.61 (CH_2_, s)	–		
37	Sucrose	342.2965	C_12_H_22_O_11_	95.1 (^1^CH)	5.42 (^1^CH, d)	3.89	√	√
				79.8 (^3′^CH)	4.22 (^3′^CH, d)	8.8		
				76.8 (^4′^CH)	4.06 (^4′^CH, t)	38.6		
				73.9 (^2^CH)	3.56 (^2^CH, m)	–		
				72.1 (^3^CH)	3.48 (^3^CH, m)	–		
				65.3 (^6^CH_2_)	3.83 (^6^CH_2_, m)	–		
				64.2 (^1′^CH_2_)	3.69 (^1′^CH_2_, s)	–		
				72.1 (^3^CH)	3.48 (^3^CH, m)	–		
38	Succinate	118.088	C_4_H_6_O_4_	37.1 (CH_2_)	2.41 (CH_2_, s)	–	√	–
39	Syringate	198.1727	C_9_H_10_O_5_	109.0 (CH)	7.28 (CH, d)	1.9	√	–
				58.7 (CH)	3.90 (CH_3_, s)	–		
40	Theophylline	180.164	C_7_H_8_N_4_O_2_	144.4 (CH)	7.98 (CH, s)	–	√	√
				32.9 (CH_3_)	3.53 (CH_3_, s)	–		
				30.8 (CH_3_)	3.35 (CH_3_, s)	–		
41	Threonine	119.1192	C_4_H_9_NO_3_	63.4 (C^α^)	3.55 (H^α^, d)	5.20	√	–
				23.0 (C^γ^)	1.33 (H^γ^, d)	6.49		
42	Trigonelline	137.136	C_7_H_7_NO_2_	148.2 (^2^CH)	9.10 (^2^CH, s)	8.80	√	√
				147.4 (^4,6^CH)	8.84 (^4,6^CH, t)	7.37		
				130.5 (^5^CH)	8.08 (^5^CH, t)			
				51.1 (^1^CH_3_)	4.42 (^1^CH_3_, s)			
43	Tyrosine	181.1885	C_9_H_11_NO_3_	38.2 (C^β^)	3.10 (H^β^, dd)	7.30, 14.30	√	–
44	Valine	117.1463	C_5_H_11_NO_2_	63.0 (C^α^)	3.60 (H^α^, d)	4.54	√	–
				20.8 (C^γ^)	0.98 (H^γ^, d)	7.10		
				19.5 (C^γ^)	1.03 (H^γ^, d)	7.10		
45	Myo-Inositol	180.1559	C_6_H_12_O_6_	77.0 (CH)	3.30 (CH, t)	–	√	√
				73.8 (CH)	3.60 (CH, dd)	4.3, 1.8		

In mature fruits, glucose and glucose-6-phosphate were the most abundant, whereas sucrose and pinitol predominated in immature fruits (Fig. S5A). This developmental shift in sugar metabolism, associated with fruit maturation, reflects a transition from sugar import and storage to enhanced glycolytic activity and metabolic readiness. While Farag et al.^22^ identified sucrose as the primary sugar in *B. aegyptiaca* fruits collected from the Red Sea Governorate in Egypt, our findings highlight distinct geographical and/or metabolic variations. The accumulation of monosaccharides in mature fruits suggests enhanced glycolytic flux, possibly supporting increased energy demand and biosynthetic activity during ripening. These differences underscore the importance of considering both developmental stage and geographic origin in metabolomic profiling of *B. aegyptiaca* fruits.

Furthermore, several organic acids were identified, with malate and citrate being the most prevalent in immature fruits, while pyruvate was the most prevalent in mature fruits (Fig. S5B). Amino acids and their derivatives were detected in the fruits, with homoserine and proline being the most abundant in both maturation stages. In mature fruits, homoserine and proline were present at concentrations of 1.4 ± 0.004 mM and 2.6 ± 0.15 mM, respectively, whereas their levels were significantly increased in immature fruits, reaching 5.5 ± 0.006 mM and 5.8 ± 0.33 mM, respectively (Fig. S5C). Histamine and phenylalanine exhibited the lowest concentrations. This finding is in contrast to Cook et al.^23^, who identified glutamic acid as the most abundant amino acid in *B. aegyptiaca* fruits from Niger.

Some aromatic amino acids, alkaloids and phenols such as theophylline and trigonelline, and syringate were identified in the aromatic region (Fig. S5D). Trigonelline has been previously reported in the fruits of *B. aegyptiaca*^22^. Notably, this study reports the first detection of theophylline and syringate in this species. These metabolites were not previously documented in *B. aegyptiaca.*

The polar extract obtained from the immature fruit was characterized by fifteen compounds, including asparagine, tyrosine, and valine, which were not detected in mature fruit. These amino acids are more abundant in early fruit development, playing a key role in protein synthesis^24^. It has been found that their concentration is significantly increased in immature tomato fruit compared to mature fruit^25^.

γ-Aminobutyrate and threonine were detected in immature fruit but were absent in mature fruit. Conversely, glutamate was present in mature fruit but not in immature fruit. Glutamate has been recognized as a key biomarker in fruit ripening. During fruit maturity, the concentration of glutamate and its derivative, γ-aminobutyrate, undergoes substantial changes. Our findings are consistent with those of Sorrequieta et al.^26^, who observed a decrease in γ-aminobutyrate levels in ripe tomato fruit and an increase in glutamate concentration. Glutamate is the most abundant amino acid in mature tomato fruit, playing a critical role in flavor development. Its high concentration contributes to the umami taste, which is a major component of the sensory profile of ripe tomatoes^26^. The ripening process has been shown to significantly elevate glutamate levels^27^. This increase in glutamate concentration is attributed to a reduction in the activity of glutamate decarboxylase, which normally converts glutamate into γ-aminobutyrate and carbon dioxide, and an enhanced activity of GABA transaminase in mature fruit^26^.

**Fig. 3:**
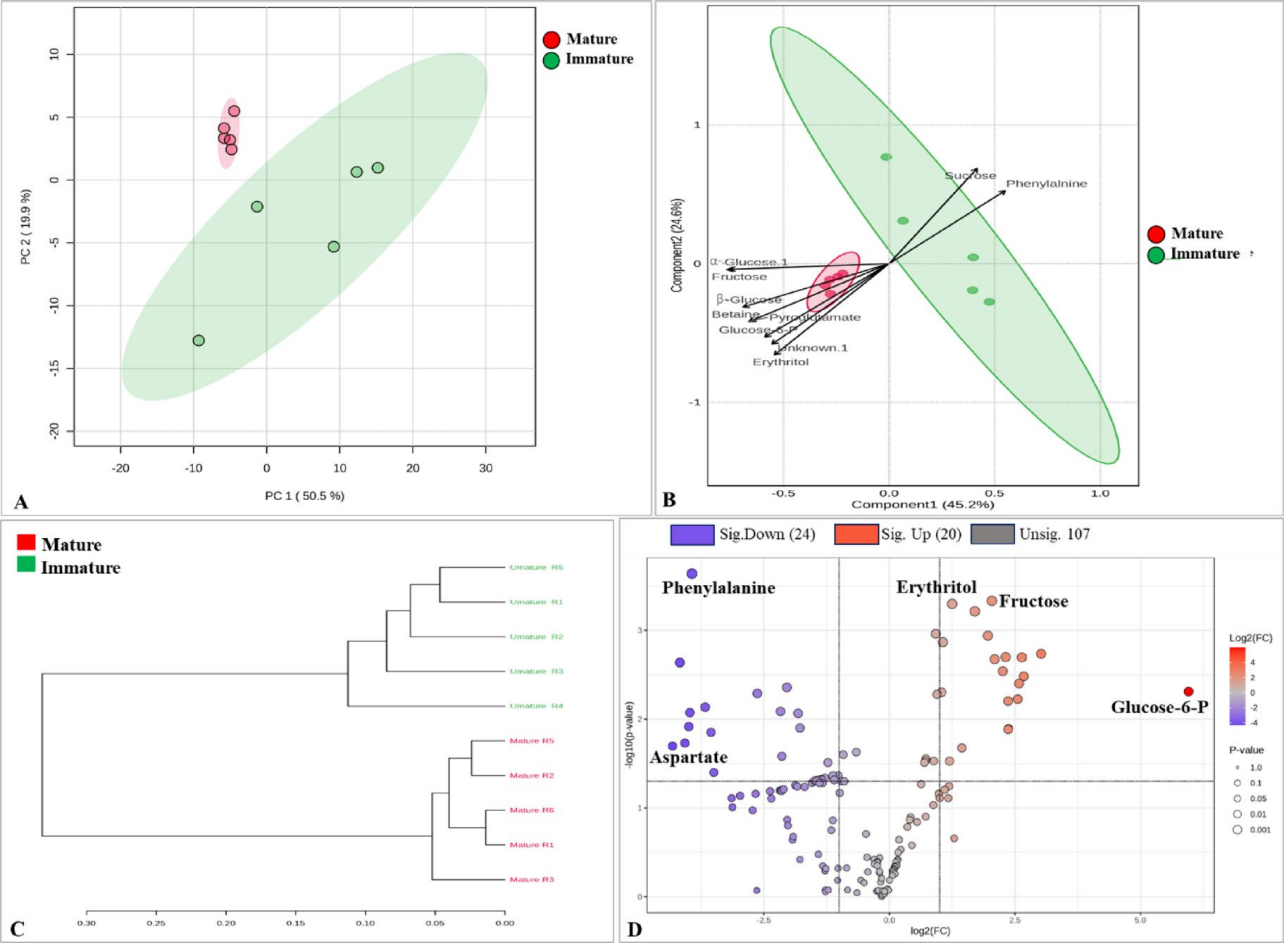
Statistical analysis of metabolomic differences between immature and mature *B. aegyptiaca* fruits. (**A**) 2D score plot of PCA showing clear separation between immature and mature fruit samples; ellipses represent 95% Hotelling’s confidence intervals, (**B**) PLS-DA biplot, (**C**) HCA dendrogram and (**D**) volcano plot identifying significantly different metabolites (Mature/Immature); the five most significant metabolites are labelled.

Multivariate analyses reveal distinct metabolic profiles between polar extracts of mature and immature fruits. In the unsupervised PCA model (Fig. 3A), the first two principal components collectively captured 70.4% of the total variance. Similarly, the supervised PLS-DA model (Fig. 3B), used to maximize class discrimination, showed that Component 1 and Component 2 accounted for 45.2% and 24.6% of the variance, respectively, confirming the distinct grouping between the two maturity stages. Also, Hierarchical clustering (Fig. 3C) further confirmed this differentiation. The PLS-DA biplot highlighted several metabolites that contributed significantly to the discrimination between groups along component 1. Among these, sucrose, phenylalanine, fructose, glucose, erythritol, betaine, and pyroglutamate were identified as key variables driving the separation, indicating their potential role in differentiating the metabolic profiles.

**Fig. 4:**
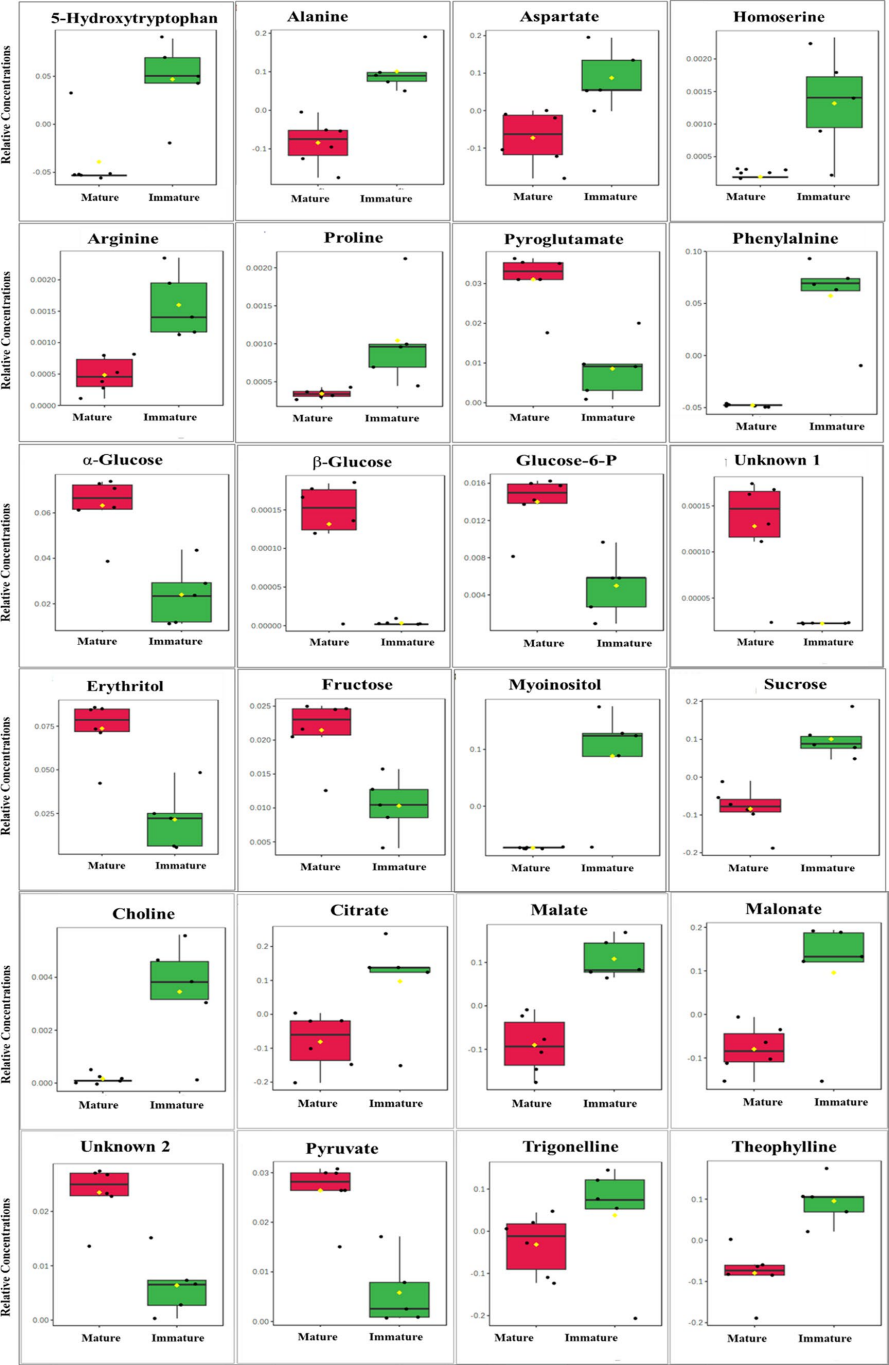
Boxplots of relative concentrations of the metabolites in polar extracts from mature and immature fruit (the 24 most significant metabolites selected from Volcano plot analysis). The black dots represent the concentration of the selected metabolite in each replicate. The mean concentration of each treatment is indicated by the yellow diamond. The Y axis defines the relative abundances of the specific metabolite, and the X axis defines the treatment group.

Furthermore, the volcano plot (Fig. 3D) was generated to identify potential biomarkers by integrating fold-change analysis and statistical significance (*p* value), with several metabolites meeting the thresholds (|log2FC|> 1, *p* < 0.05). This approach enables the identification of metabolites that are not only statistically significant but also biologically relevant to the maturation process. A total of 24 metabolites were upregulated and 20 were downregulated during fruit maturation. The box plots (Fig. 4) generated based on the volcano plot analysis show the relative concentrations of the most significant metabolites.

The box plots show a higher concentration of sucrose coupled with a lower concentration of monosaccharides in immature fruit compared to mature fruit indicating the accumulation of simple sugars and a change in metabolism during ripening. This plays a role in enhancing the sweetness and flavor of the fruit^28^. The increase in monosaccharides and decrease in disaccharides in mature fruit is attributed to increased activity of hydrolytic enzymes, such as invertase and amylase. These enzymes exhibit higher activity in ripe compared to unripe fruit^29,30^. Erythritol is a sugar alcohol known for its pleasant sweetness, utilized as a sweetener in calorie-reduced foods^31^. This metabolite is characterized by several biological activities, including antidiabetic and antioxidant properties, and it can reduce plaque and potentially prevent decay of teeth^32,33^.

Phenylalanine, a precursor in the phenylpropanoid pathway, plays a critical role in synthesizing secondary metabolites and defense-related compounds in pear fruit^34^. It has been also identified as a key contributor to the volatile aroma in plants^35,36^. Betaine has a role in maintaining the osmotic balance and protecting the cellular structure during stress conditions^37^. Earlier studies have demonstrated that betaine affects the antioxidant and membrane fatty acid metabolism of fruits and vegetables to mitigate abiotic stress damage^38^. It has been reported to improve the flavor quality of peach fruit^39^.

Based on box plot analysis, the relative quantities of amino acids arginine, proline, phenylalanine, and homoserine, were higher in immature fruit compared to mature fruit. Amino acids serve as building blocks of proteins and precursors for various secondary metabolites which are necessary for fruit maturation and protection under stress conditions. Arginine plays a crucial role in both plant development and stress tolerance. As a nitrogen-rich amino acid, arginine serves as a key nitrogen storage molecule and a precursor for polyamines, which are essential for plant growth and fruit ripening^40^. It also contributes to abiotic stress responses as a precursor of polyamines and nitric oxide, which help to stabilize membranes, and to enhance the antioxidant defense mechanisms under drought, salinity, and oxidative stresses^40,41^. Proline is known for its role in osmoprotection in mitigating abiotic stresses such as drought and high temperatures common in arid regions^42^. Phenylalanine serves as a pivotal precursor in the biosynthesis of various aroma compounds in fruits through the phenylpropanoid pathway. This pathway leads to the formation of volatile phenylpropanoids which contribute to the characteristic floral and fruity aromas of many fruits^35^.

Herein, the concentrations of organic acids citrate and malate, were found to be higher in immature fruit. Citrate and malate have been identified as the predominant organic acids in a variety of fruits^43,44^. Citric acid and malic acid are known to accumulate in larger concentrations in immature fruit and serve as respiratory substrates throughout fruit ripening. Consequently, their concentrations decrease during the last stages of fruit maturation^45^. Passion fruit exhibited the highest levels of citric acid during its immature stage, which decreased as the fruit matured^46^. During grape ripening, malate released from the vacuole plays a pivotal role as the primary carbon source for energy metabolism and biosynthesis, replacing sugars.

The increased concentrations of theophylline and trigonelline in immature fruit in the present study are consistent with the findings of Pereira et al.^30^, who observed a reduction in alkaloid levels with fruit development. According to Koshiro et al.^47^, trigonelline exhibited more biosynthetic activity in young fruit. As the fruit matures, trigonelline is transported from the outer layer to the seeds. This process explains the drop in trigonelline concentration observed in mature fruit. Alkaloids are known to play a significant role in plant defense mechanisms by deterring herbivores and inhibiting microbial growth, which is particularly important during the vulnerable early stages of fruit development^48^.

### Metabolic pathway changes during fruit maturation

**Fig. 5:**
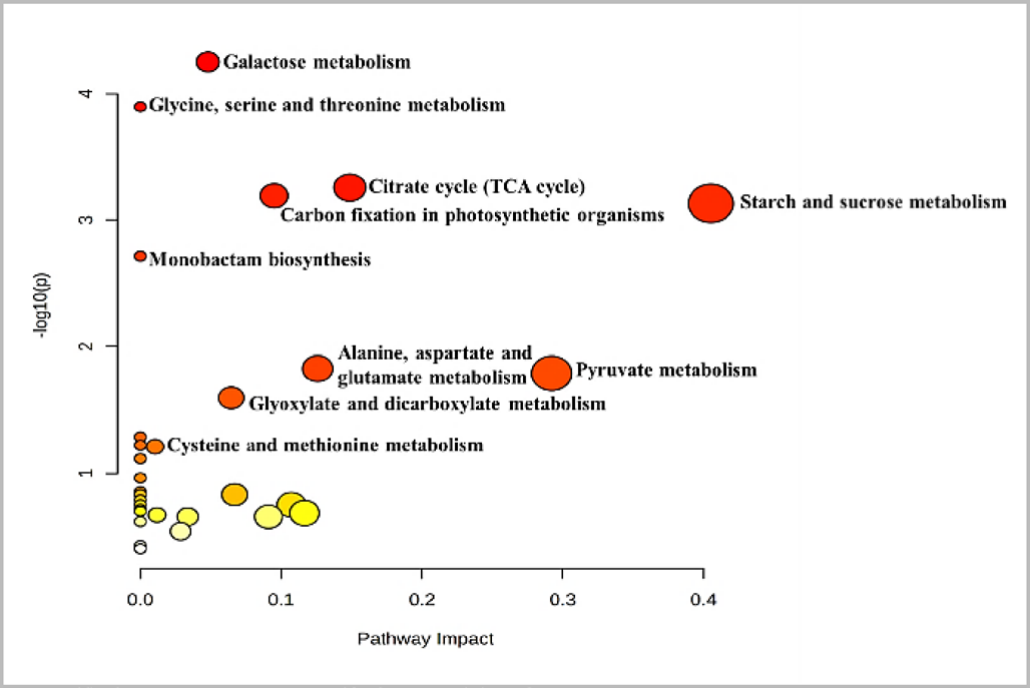
Metabolic pathways in *B. aegyptiaca* that are significantly altered between mature and immature fruit based on KEGG database (www.kegg.jp/kegg/kegg1.html). The dark red circles indicate the pathways that were significantly affected. As the *p* value increases, the color progressively fades, whereas the larger the circle size, the greater the pathway’s influence.

**Fig. 6:**
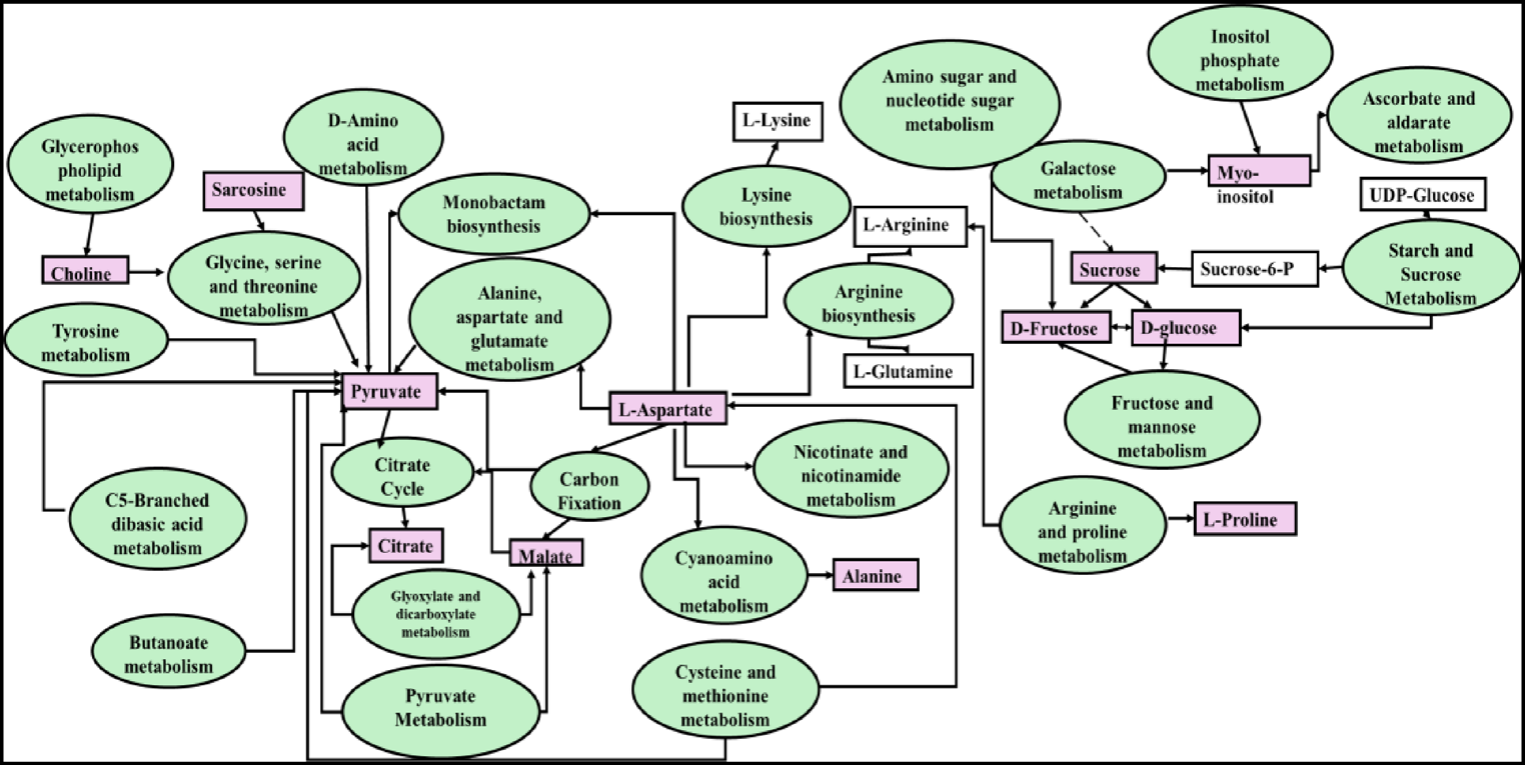
Metabolic pathways that significantly changed (in green ovals) as well as the significant metabolites (in pink squars) that changed between immature and mature *B. aegyptiaca* fruits.

Significant alterations in metabolic pathways during the ripening of *B. aegyptiaca* fruit are illustrated in Fig. 5. During the transition from the immature to the mature stage, a total of thirty-nine metabolic pathways exhibited significant changes based on the pathway analysis (Table 2). Starch and sucrose metabolism, pyruvate metabolism, and the citrate cycle (TCA) pathways had particularly strong impacts between mature and immature fruit. The relationship between the significant metabolic pathways and the significant metabolites is shown in Fig. 6. The starch–sucrose metabolism pathway is of great importance among the many metabolic pathways involved in fruit ripening. This pathway controls the process of converting starch into sucrose and then to the monosaccharides glucose and fructose, which are crucial for the taste, sweet flavor, and overall fruit quality^49,50^. Moreover, the starch–sucrose metabolism pathway is intricately linked to sugar signaling mechanisms that regulate gene expression and enzymatic activities during fruit ripening^51,52^. Sugar signals can induce the expression of ethylene biosynthesis genes, thereby enhancing the ripening process^52^.

**Table 2:** Pathway analysis depicting significantly altered metabolic pathways in *Balanites aegyptiaca* fruit extracts.

Pathway	Total	Expected	Hits	Raw p	Holm adjust	FDR	Impact
Carbon fixation	21	0.19626	4	2.62E−05	0.002433	0.002433	0.09578
Galactose metabolism	27	0.25234	4	7.43E−05	0.006833	0.003453	0.04805
Glycine, serine and threonine metabolism	33	0.30841	4	0.000168	0.015254	0.005196	0
Citrate cycle (TCA cycle)	20	0.18692	3	0.000675	0.060789	0.013987	0.15879
Alanine, aspartate and glutamate metabolism	22	0.20561	3	0.000902	0.08031	0.013987	0.1259
Starch and sucrose metabolism	22	0.20561	3	0.000902	0.08031	0.013987	0.40507
Monobactam biosynthesis	8	0.074766	2	0.002201	0.19145	0.029236	0
Pyruvate metabolism	23	0.21495	2	0.01835	1	0.21332	0.29205
Glyoxylate and dicarboxylate metabolism	29	0.27103	2	0.02852	1	0.2947	0.15112
C5-Branched dibasic acid metabolism	6	0.056075	1	0.05487	1	0.51029	0
D-Amino acid metabolism	7	0.065421	1	0.063739	1	0.5147	0
Cysteine and methionine metabolism	46	0.42991	2	0.066413	1	0.5147	0.01038
Lysine biosynthesis	9	0.084112	1	0.081245	1	0.58121	0
Selenocompound metabolism	13	0.1215	1	0.11535	1	0.67728	0
Nicotinate and nicotinamide metabolism	13	0.1215	1	0.11535	1	0.67728	0
Butanoate metabolism	17	0.15888	1	0.14827	1	0.67728	0
Arginine biosynthesis	18	0.16822	1	0.15632	1	0.67728	0
Tyrosine metabolism	18	0.16822	1	0.15632	1	0.67728	0
beta-Alanine metabolism	18	0.16822	1	0.15632	1	0.67728	0
Fructose and mannose metabolism	18	0.16822	1	0.15632	1	0.67728	0.06695
Ascorbate and aldarate metabolism	20	0.18692	1	0.17222	1	0.67728	0
Cyanoamino acid metabolism	21	0.19626	1	0.18006	1	0.67728	0
One carbon pool by folate	21	0.19626	1	0.18006	1	0.67728	0
Thiamine metabolism	22	0.20561	1	0.18783	1	0.67728	0
Valine, leucine and isoleucine biosynthesis	22	0.20561	1	0.18783	1	0.67728	0.10727
Lipoic acid metabolism	24	0.2243	1	0.20317	1	0.67728	0
Tryptophan metabolism	25	0.23364	1	0.21074	1	0.67728	0
Pantothenate and CoA biosynthesis	25	0.23364	1	0.21074	1	0.67728	0
Glycolysis or Gluconeogenesis	26	0.24299	1	0.21824	1	0.67728	0.11665
Glutathione metabolism	27	0.25234	1	0.22567	1	0.67728	0.01195
Arginine and proline metabolism	28	0.26168	1	0.23304	1	0.67728	0.03364
Inositol phosphate metabolism	28	0.26168	1	0.23304	1	0.67728	0.08958
Terpenoid backbone biosynthesis	31	0.28972	1	0.25476	1	0.71796	0
Glycerophospholipid metabolism	38	0.35514	1	0.30324	1	0.82945	0.02862
Amino sugar and nucleotide sugar metabolism	52	0.48598	1	0.39153	1	1	0

The table shows the detailed results from the pathway analysis generated with MetaboAnalyst 6.0. Since many pathways are being tested at the same time, the statistical *p* values from enrichment analysis are further adjusted for multiple testings. In particular, *Total* is the total number of compounds in the pathway; *Hits* is the matched number from the user uploaded data; *Raw p* is the original p value calculated from the enrichment analysis; *Holm p* is the p value adjusted by Holm-Bonferroni method; *FDR p* is the p value adjusted using False Discovery Rate; *Impact* is the pathway impact value calculated from pathway topology analysis.

The pyruvate metabolism pathway plays a central role in fruit maturity. As an end-product of glycolysis, pyruvate fuels the TCA cycle for ATP generation and organic acid metabolism, influencing fruit flavor. It also serves as a precursor for amino acids. Pyruvate serves as a precursor of acetaldehyde by the enzyme pyruvate decarboxylase, a natural aromatic component found in nearly all fruits, which accumulates throughout ripening. Acetaldehyde is a precursor to other naturally aromatic compounds^49^. Moyano et al.^50^ investigated the roles of two pyruvate decarboxylase genes (Fapdc1 and Fapdc3) in strawberry fruits. The former was reported as crucial for fruit ripening and aroma biosynthesis, while the latter is implicated in overall metabolism to facilitate energy production and the biosynthesis of more complex metabolites.

The TCA cycle is a key metabolic pathway responsible for the production of organic acids and secondary metabolites that contribute to the color, taste, and flavor of mature fruit compared to immature fruit. The use of TCA flow in energy production or metabolite biosynthesis is determined by the cell’s energy needs and physiological state^53–55^. According to a study by Tao et al.^56^ and Li et al.^57^, the TCA cycle was the predominant pathway involved in the maturation process of apple and blueberry fruit.

### Cytotoxic activity of the fruit extracts against hepatocellular carcinoma

**Fig. 7:**
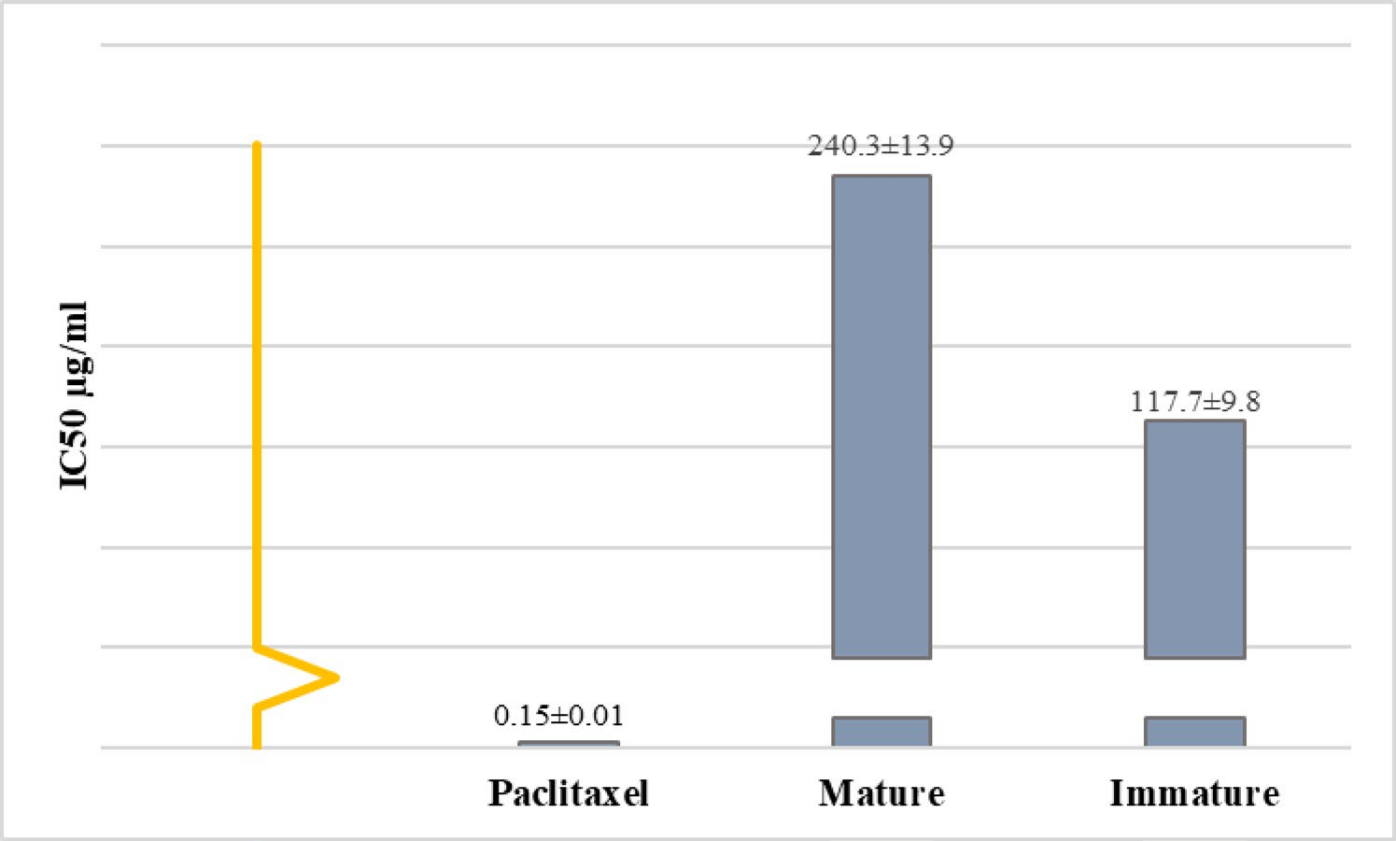
IC50 values show the effect of *B. aegyptiaca* fruit extracts on cell viability of hepatocellular carcinoma.

The results showed that the polar extracts of immature fruit exhibited better cytotoxic activity against hepatocellular carcinoma compared to the mature fruit, with IC_50_ values of 117.7 µg/mL and 270.4 µg/mL, respectively (Figs. 7 and S7). The IC_50_ value for the positive control, paclitaxel, was 0.15 µg/mL. Fig. S6 illustrates the morphological characteristics of cells observed under inverted microscopy. Untreated control cells exhibit a dense, compact, and well-organized structure, indicative of typical carcinoma morphology. In contrast, cells treated with paclitaxel show pronounced cytotoxic effects, including cell shrinkage, structural distortion, and markedly reduced cell density. Exposure to the polar extract of immature fruits results in significant morphological alterations, characterized by considerable structural disruption and a decrease in cell density. In comparison, cells treated with the polar extract of mature fruits lower levels of cytotoxicity, indicating a reduced antiproliferative effect relative to the immature fruit extract.

The extracts from both developmental phases are rich in bioactive metabolites with well-documented roles in cancer treatment, such as theophylline^58^, trigonelline^59,60^, phenylalanine^61^, histamine^62^, arginine^63^, and d-pinitol^64,65^. We propose that the pronounced cytotoxic effects of the polar extract obtained from immature fruit, compared to the extract derived from mature fruit, are due to the higher concentrations of these metabolites in immature fruit. Moreover, the extract of immature fruit showed the presence of fifteen metabolites, many of which are well known to possess biological activities. For example, syringate has anti-inflammatory, antioxidant, and antimicrobial activities^66^.

### In silico study

**Fig. 8:**
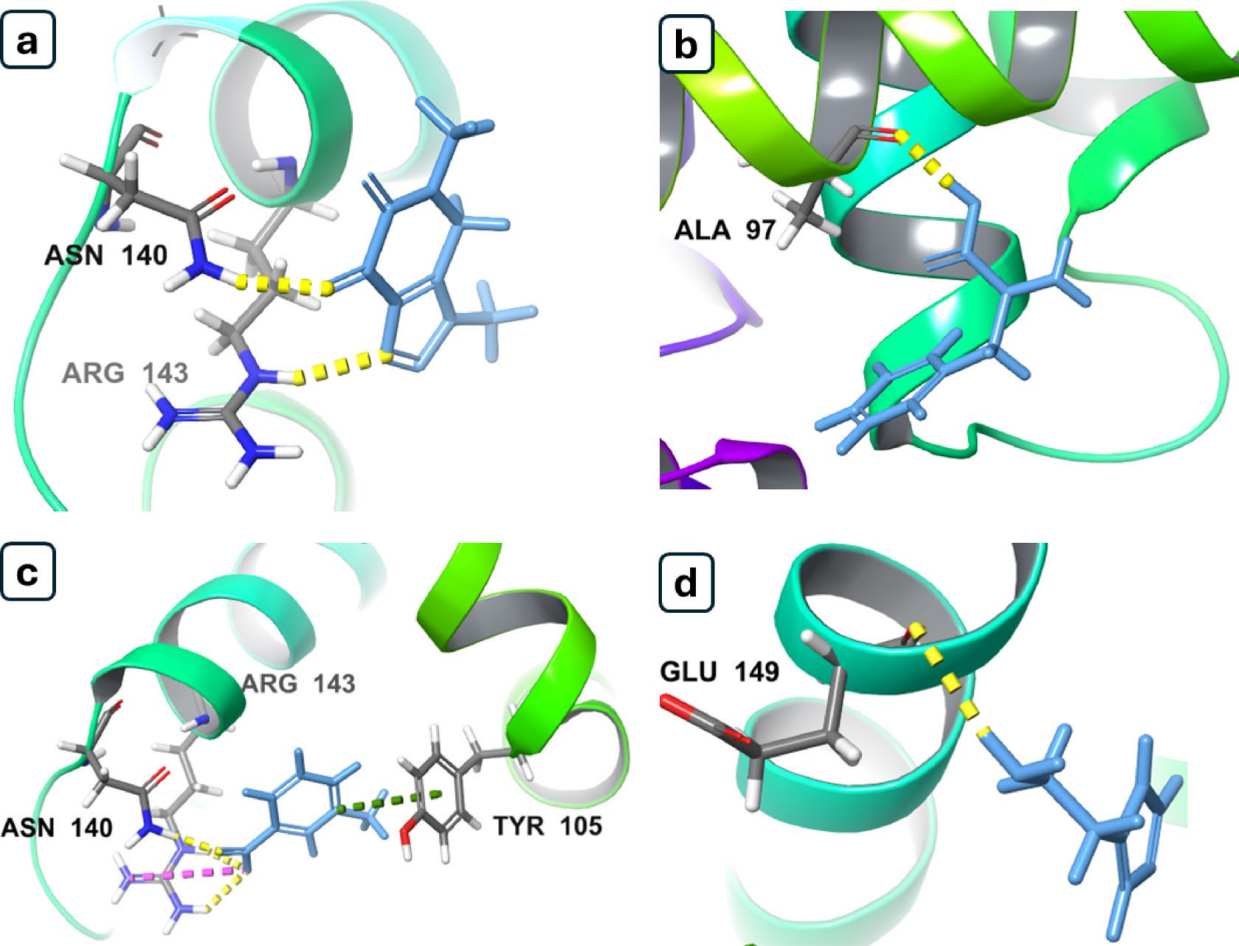
Binding modes of top compounds from *B. aegyptiaca* fruit extracts against BCL-2 protein: (**a**) theophylline (both immature and mature), (**b**) phenylalanine (immature), (**c**) trigonelline (both immature and mature), (**d**) histamine (immature).

The molecular docking study demonstrated that 29 metabolites from *B. aegyptiaca* fruit extracts interacted with the BCL-2 protein (PDB ID: 4LVT), supporting their potential cytotoxic activity. The binding poses of some compounds are visualized in Fig. 8. Theophylline exhibited the highest docking score (− 5.317 kcal/mol), forming hydrogen bonds with ASN 140 and ARG 143 (Table S1), consistent with its pro-apoptotic effects^67^. Phenylalanine, detected in immature extracts, scored − 4.657 kcal/mol with a hydrogen bond to ALA 97, potentially contributing to cytotoxicity via dipeptide derivatives. Trigonelline (− 4.652 kcal/mol) formed a hydrogen bond with ASN 140, a salt bridge with ARG 143, and a π–cation interaction with TYR 105, aligning with its anticancer properties^59,60^. Histamine (− 4.649 kcal/mol), also in immature extracts, formed a hydrogen bond with GLU 149, suggesting receptor-mediated anticancer effects^68^. Other compounds, such as asparagine (− 2.992 kcal/mol), may influence cancer cell survival^69^. These findings highlight the superior bioactivity of immature fruit extracts compared to mature extracts.

The integration of metabolomics, in vitro cytotoxicity assays, and in silico molecular docking provides a powerful, multi-layered approach to understanding the therapeutic potential of *B. aegyptiaca* fruits. This study moves beyond merely cataloging the chemical constituents to functionally connecting them with biological activity. First, the identification of distinct metabolic profiles of immature and mature fruits established a chemical basis for their differential effects. The subsequent cytotoxicity assays confirmed that the higher concentration of certain alkaloids, organic acids, and amino acids in immature fruits correlates with enhanced anticancer efficacy. The in silico docking analysis provides crucial mechanistic insight, suggesting that these specific metabolites can directly interact with and inhibit key anti-apoptotic proteins like BCL-2. This synergistic approach, linking chemical composition to cellular effect and then to molecular target interaction, validates the superior therapeutic potential of immature fruits and provides a robust framework for future drug discovery efforts based on these natural products.

## Conclusion

This study presents the first in-depth metabolomic investigation of *B. aegyptiaca* fruit maturation using advanced 1D and 2D NMR data, leading to the identification of 45 metabolites and significant metabolic transitions. Multivariate statistical analysis revealed clear metabolic differences between immature and mature stages. Key metabolites that characterized each stage were successfully identified. Immature fruits were characterized by elevated levels of sucrose, pinitol, malate, and citrate, whereas mature fruits showed an increase in monosaccharides and pyruvate, reflecting a metabolic shift favoring sugar hydrolysis and organic acid utilization. Pathway analyses highlighted key alterations in starch–sucrose and pyruvate metabolism, offering insights into biochemical adaptations in desert-adapted species. Notably, immature fruit extracts demonstrated higher cytotoxic activity against hepatocellular carcinoma than mature fruit. Molecular docking confirmed the interactions of some of the identified metabolites with the anti-apoptotic BCL-2 protein, supporting their apoptotic potential.

## Supplementary Information

Below is the link to the electronic supplementary material.

Supplementary Material 1

## Data Availability

The datasets used and/or analyzed during the current study are available from the corresponding author upon reasonable request.
