# Supplementary material for: Maturity related metabolomic analysis of Balanites aegyptiaca fruits with in vitro and in silico cytotoxicity evaluation
Source: Sci Rep. 2025 Aug 23;15:31035. doi: 10.1038/s41598-025-15234-y (PMC12375094; doi:10.1038/s41598-025-15234-y)
Supplement: Supplementary file 1 — Supplementary Material 1 [file 41598_2025_15234_MOESM1_ESM.docx]

**Supplementary Material**

**Maturity-related metabolomic analysis of *Balanites aegyptiaca* fruits with *in vitro* and *in silico* cytotoxicity evaluation**

**Asmaa Abdelsalam^*1, †^, Ehab Mahran ^2,†^, Eslam T Mohamed^1^,** **Arezue Boroujerdi^3^, Hebatallah Aly^1^**

**Affiliation**

^1^ Botany Department, Faculty of Science, Helwan University, Cairo 11795, Egypt.

^2^ CAMAG Chemical Products and Adsorption Technology AG, Muttenz 4132, Switzerland.

^3^ Chemistry Department, Claflin University, Orangeburg, SC 29115, USA.

† These authors contributed equally to this work.


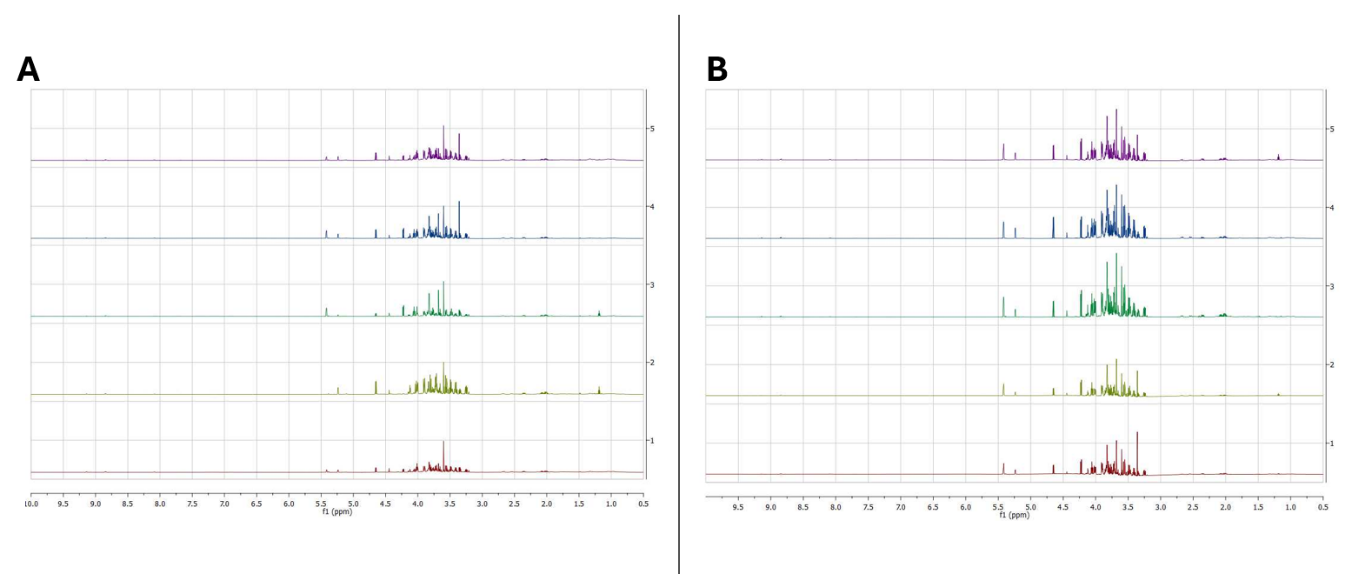


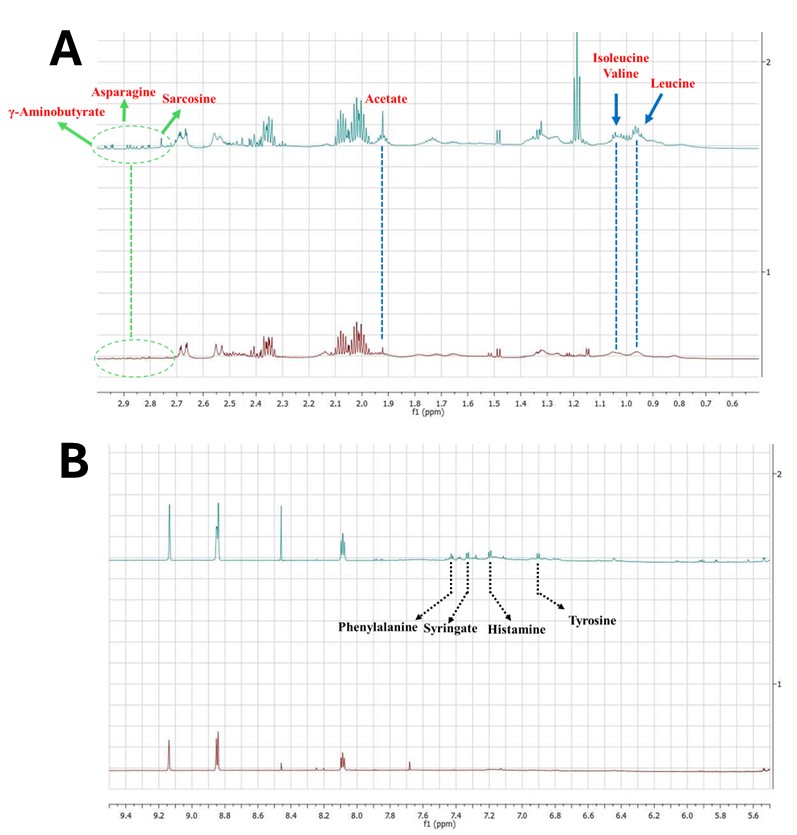
**Fig. S1** ^1^H NMR spectra (n = 5) of the polar extracts of the immature (**A**) and mature (**B**) fruit.

**Fig. S2** ^1^H NMR spectra of Balanites aegyptiaca polar extract in the region δ_H_ 0.5 – 3.0 ppm (**A**) and δ_H_ 5.5 – 10.0 ppm (**B**). Spectra of the immature fruit (top) and mature fruit (bottom).


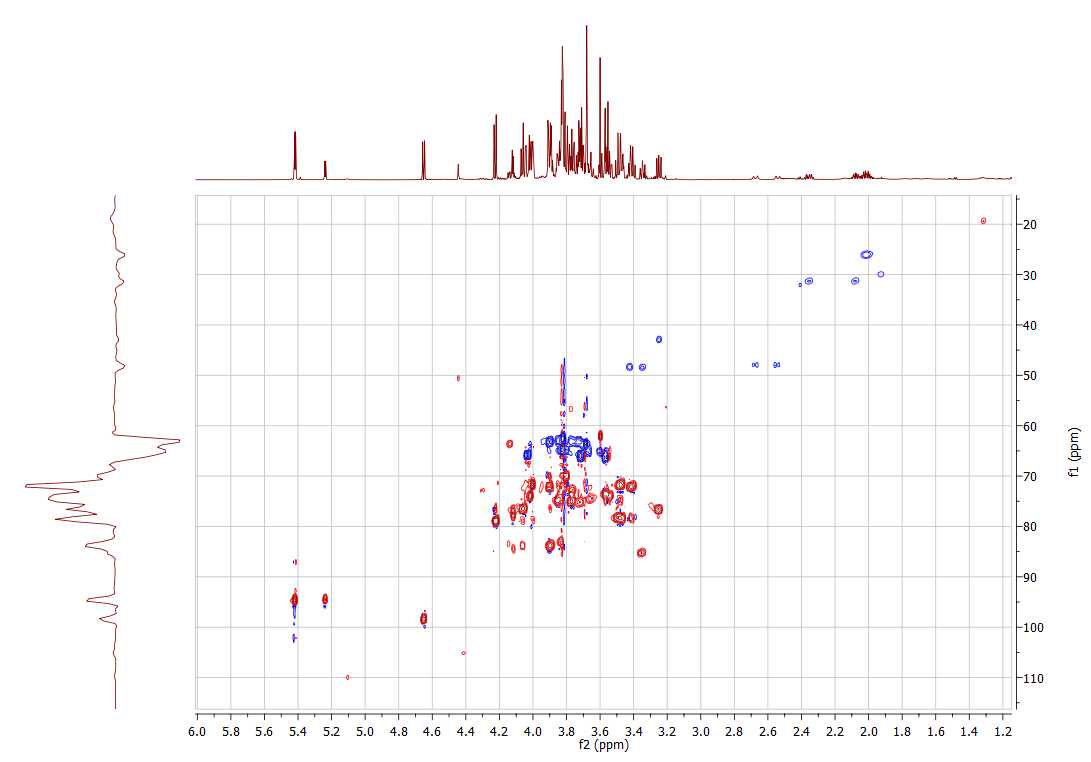


**Fig. S3** ^1^H-^13^C HSQC spectrum of Balanites aegyptiaca polar extract. f2 = δ_H_ and f1 = δ_C_.


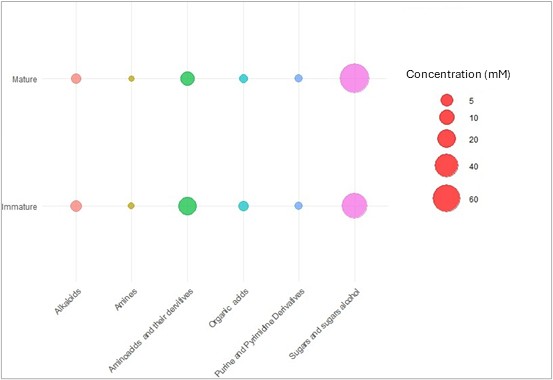


**Fig. S4** Concentrations of different chemical groups identified in mature and immature fruits of Balanites aegyptiaca polar extract.


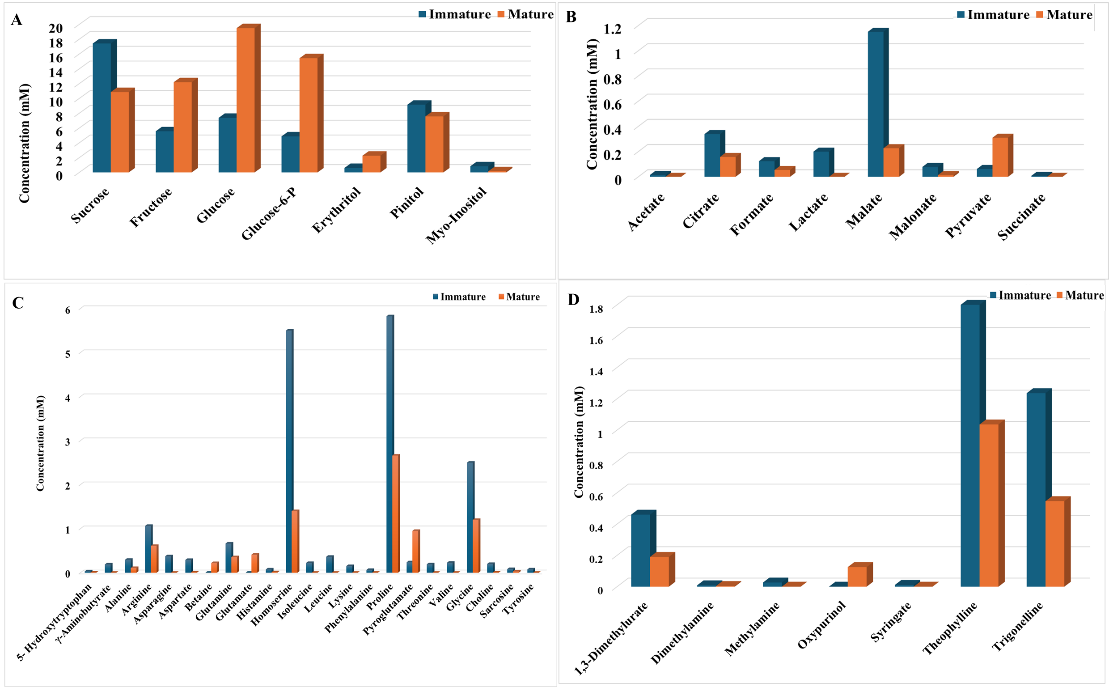


**Fig. S5** The mean concentrations of (**A**) sugars and sugars alcohol; (**B**) organic acids; (**C**) amino acids and amino acids derivatives and (**D**) phenols, alkaloid, purine and pyrimidine derivatives in the polar extracts of Balanites aegyptiaca.

**
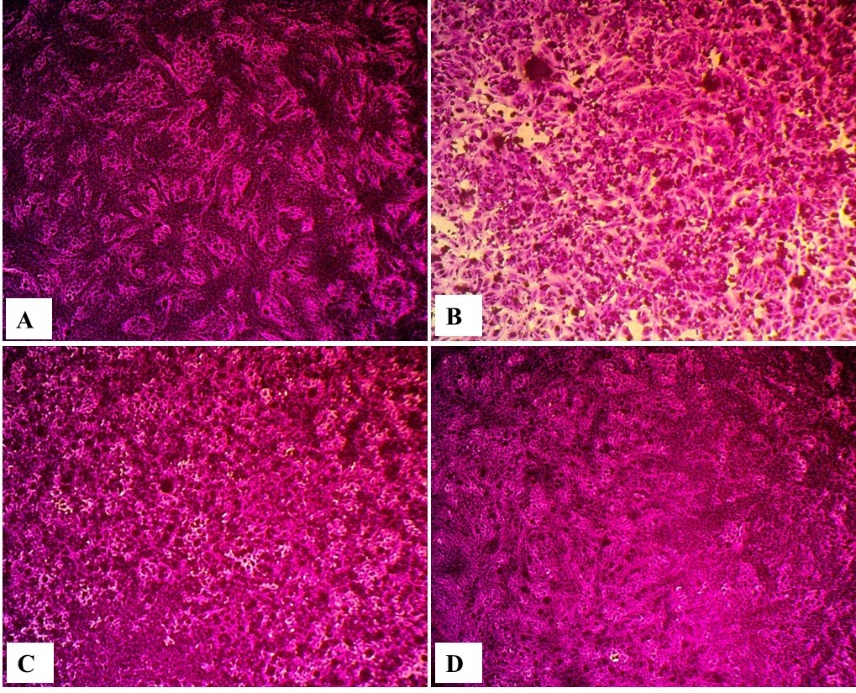
**

**Fig. S6** The inverted microscope images of hepatocellular carcinoma (**A**) control (untreated cells); (**B**) treated with Paclitaxel, (**C**) treated with immature fruits polar extract and (**D**) treated with mature fruits polar extract. Magnification 100x.


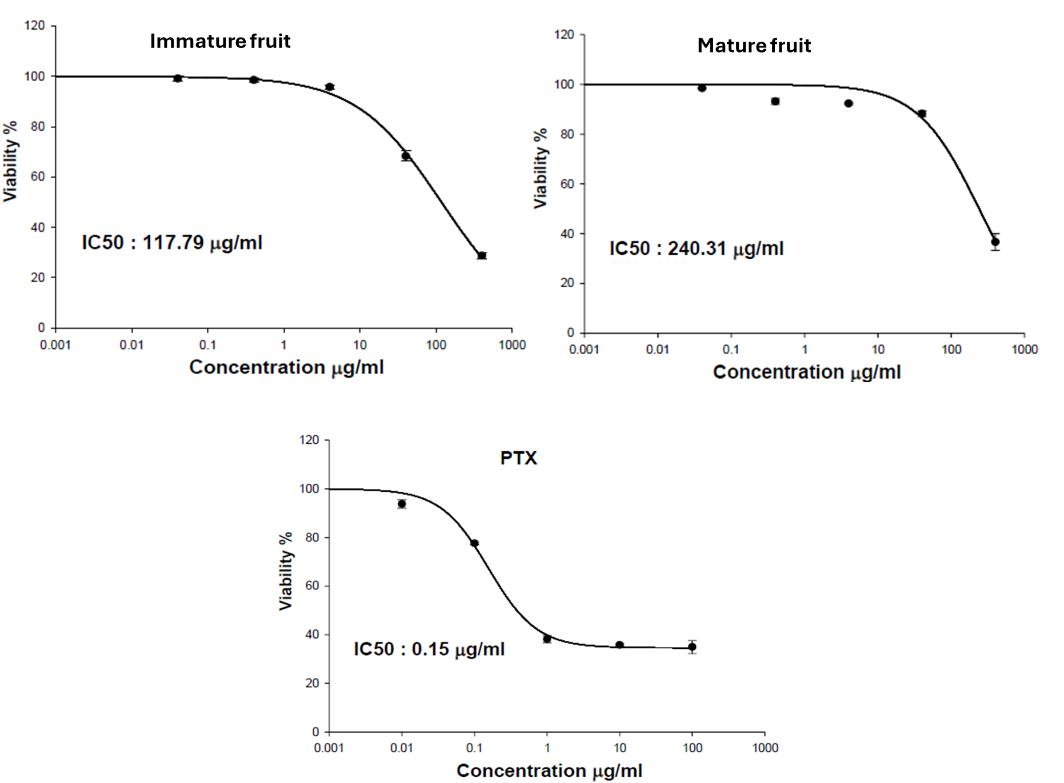


**Fig. S7** The effect of *B. aegyptiaca* fruit extracts on cell viability of hepatocellular carcinoma.

**Table S1** Docking interactions of selected compounds with BCL-2 protein.

| **Compound Name** | **Fruit Maturity Source** | **Docking Score (kcal/mol)** | **Interaction Type** | **Residues Involved** |
| --- | --- | --- | --- | --- |
| Theophylline | Both | −5.317 | Hydrogen bond | ASN 140, ARG 143 |
| Phenylalanine | Immature | −4.657 | Hydrogen bond | ALA 97 |
| Trigonelline | Both | −4.652 | Hydrogen bond, Salt bridge, π-cation | ASN 140, ARG 143, TYR 105 |
| Histamine | Immature | −4.649 | Hydrogen bond | GLU 149 |
| Syringate | Immature | −4.513 | None | — |
| Alanine | Both | −4.400 | None | — |
| Valine | Immature | −4.283 | Hydrogen bond | ALA 146 |
| Myo-Inositol | Both | −4.261 | Hydrogen bond | ASP 100 |
| Proline | Both | −4.441 | None | — |
| 5-Hydroxytryptophan | Both | −4.377 | Hydrogen bond | ASP 108, ASN 140 |
| Tyrosine | Immature | −3.812 | Hydrogen bond, π-π stacking | ASP 108, PHE 101 |
| Sucrose | Both | −3.622 | Hydrogen bond | LEU 134, ASP 137, ARG 143 |
| Malonate | Both | −3.752 | None | — |
| Lactate | Immature | −3.632 | Hydrogen bond, Salt bridge | ASN 140, ARG 143 |
| Leucine | Immature | −3.607 | Hydrogen bond | ALA 97 |
| Acetate | Immature | −3.530 | None | — |
| Methylamine | Immature | −3.446 | None | — |
| Choline | Both | −3.458 | None | — |
| Isoleucine | Immature | −3.277 | Hydrogen bond | GLU 142 |
| Citrate | Both | −3.260 | Hydrogen bond, Salt bridge | ASN 140, ARG 143 |
| Malate | Both | −3.184 | Hydrogen bond, Salt bridge | ASN 140, ARG 143 |
| Succinate | Immature | −3.113 | Hydrogen bond, Salt bridge | ASN 140, ARG 143 |
| Aspartate | Both | −3.174 | Hydrogen bond, Salt bridge | ARG 144 |
| Asparagine | Immature | −2.992 | Hydrogen bond | ASP 108 |
| Sarcosine | Immature | −2.690 | Hydrogen bond | ASP 108 |
| Arginine | Both | −2.652 | Hydrogen bond | LEU 134, ASP 137, ASN 140 |
| Homoserine | Both | −2.460 | Hydrogen bond | ALA 146, GLU 149 |
| Lysine | Immature | −2.187 | Hydrogen bond | ALA 97, GLU 142 |
| γ-Aminobutyrate | Immature | −1.767 | Hydrogen bond | ASP 108, GLU 142 |
